# How Do Legal Experts Cope With Medical Reports and Forensic Evidence? The Experiences, Perceptions, and Narratives of Swiss Judges and Other Legal Experts

**DOI:** 10.3389/fpsyt.2019.00018

**Published:** 2019-02-13

**Authors:** Carlos Canela, Anna Buadze, Anish Dube, Christian Jackowski, Ingo Pude, Romilda Nellen, Paola Signorini, Michael Liebrenz

**Affiliations:** ^1^Department of Forensic Psychiatry, Institute of Forensic Medicine, University of Bern, Bern, Switzerland; ^2^Department of Psychiatry, Psychotherapy and Psychosomatics, Psychiatric Hospital, University of Zurich, Zurich, Switzerland; ^3^Department of Psychiatry and Human Behavior, UC Irvine Medical Center, Orange, CA, United States; ^4^Department for Forensic Medicine and Imaging, Institute of Forensic Medicine, University of Bern, Bern, Switzerland; ^5^Forensic Assessment Clinic, Psychiatric Hospital Münsterlingen, Münsterlingen, Switzerland; ^6^Department of Forensic Psychiatry, Luzerner Psychiatrie, Lucerne, Switzerland

**Keywords:** interdisciplinary learning, law, medical knowledge acquisition, scientific literacy skills, qualitative research, expert witness

## Abstract

Expert scientific knowledge, including medical knowledge, is relevant for the legal profession and can strongly influence rulings and sentencing in criminal law, civil law, and insurance law. The way in which this medical evidence is understood and evaluated thus has an impact both on individuals and on society as a whole. It enters legal procedures in various forms, for example, as expert witness statements and/or as a legal expert's own acquired medical knowledge. On the other hand, a legal expert may be confronted with expert medical opinions that differ in quality or content and thus have to decide which ones to follow. The aim of this qualitative study was to investigate legal experts' perceptions, experiences, and narratives regarding medical knowledge, particularly the skills and general knowledge used in their branch of the legal profession. A total of 51 semi-structured interviews with judges and prosecutors from different courts of law and from the public prosecutor's office in six different German-speaking (Zurich, Luzern, Aagrau, Obwalden, Nidwalden, Zug) and German/French-speaking (Bern) cantons of Switzerland were conducted, coded, and analyzed using Nvivo. We used a comparison thematic approach identifying common and new themes related to the research aims. Our findings suggest that Swiss judges and prosecutors believe that possessing and developing the skills and terminology required for processing medical information is important but complex, and time-consuming for their work. Additionally, several legal experts reported that their understanding of medical evidence was limited or even non-existent. Moreover, the acquisition of skills related to the assessment of medical reports and forensic evidence appeared to be unstructured. Participants reported having no formal instruction in how to evaluate or deal with medical knowledge. The sources they used to answer questions arising appeared to be in part problematic and non-standardized (internet, newspapers, etc.). Medical literature from peer-reviewed journals was used only rarely. The findings from this study suggest that law departments might wish to evaluate whether their graduates are sufficiently equipped with scientific literacy skills and appropriate skills to evaluate medical information for their later careers. At the same time, medical knowledge pertinent to forensics published in local legal journals may be more effective in reaching the legal expert audience than in medical journals.

## Highlights

- Legal experts indicated that the medical knowledge and scientific literacy skills taught during law school were insufficient, even though they often have to deal with medical information in their profession.
- After graduation medical knowledge is generally acquired by informal learning from medical expert testimony, from acquaintances with a medical background, on the job, and from the internet.
- Several barriers to the acquisition of skills to evaluate medical knowledge were identified.
- Specifically targeted ways of transferring scientific literary skills and evaluating medical knowledge are needed.

## Introduction

Modern justice systems play a crucial role in society. To fulfill their integrating role, they arguably require high-quality evidence. Legal experts and jurors are often faced with cases that need specialized knowledge outside their regular education, such as medical or other scientific information, toxicology, ballistics, or engineering (1–4). This specialized knowledge is the result of professional education and years of experience. The knowledge gap between legal experts and science experts may be exacerbated by the fact that legal methodologies and thinking differ from the methodology of natural sciences like medicine (5). Similarly, scientific jargon creates particular concepts and definitions for terms that might not coincide with those used by a layman or legal expert. A clear example is the difference between the legal and scientific notion of evidence and truth.

Medical or scientific knowledge may enter a legal process in various ways, be it in the form of an expert statement, e.g., from the treating physician, an expert hired by one of the parties or an impartial agency like the court (6), or in the form of knowledge legal experts have acquired by themselves (e.g., in books, journal articles, internet posts) (7), thus bridging the knowledge gap—at least in theory. In the case of criminal law and mental health, a mental health expert's statement could be used to exculpate an individual or lead to confinement in an institution.

Whilst in insurance law, legal decisions based on medical experts' statements may, for example, influence compensation claims for certain illnesses (1). The effect of expert testimony on rulings is complex. It is mediated by factors related to the scientific information presented, the medical expert, and the recipient (8). Since such rulings have an impact on individuals and sometimes also on society as a whole (7, 9–13), it is of paramount importance that the medical knowledge on which these decisions hinge be high-quality and comprehensive.

Unfortunately, conflicting expert testimonies are not a rarity (14), even among highly trained experts or treating physicians who have known their patients for a long time. This is particularly pertinent in the realms of psychiatry and psychology. Forensic psychiatric data and the conclusions drawn are relatively complex compared to those from other medical disciplines because biological objective markers for psychiatric assessments do not yet exist. Usually, psychiatric assessments are based on symptoms reported by patients and the results of psychological tests and behavioral observations, the latter often relying heavily on the accounts provided by the legal client/patient (15). Furthermore, the divergent assessment of even small psychopathological anomalies by different experts can have a decisive effect for the individual if psychiatric diagnoses are framed in a legal context, thus forming the basis for either a custodial prison sentence, inpatient treatment in a forensic hospital or life-long detention.

The nature of the data: how it is collected, for whom it is collected and for what purposes can increase the risks of bias and unreliability. Although studies to further our understanding of biasability and reliability of psychiatric and psychological forensic evidence are important, such efforts are infrequent and methodologically difficult compared to studies in other fields of medical forensic science (16).

Publications addressing the potential for errors in expert testimonies (17–22) and other quality problems (23–26) by research groups from different countries indicate that this widespread problem may lead to differing testimonies. In practice, it is therefore necessary for legal experts to be able to deal with differing medical expert opinions and to decide which to admit and which to follow.

There are different approaches to this problem. One well-known approach comprises defining criteria to help determine the quality of scientific evidence and its admissibility for a legal case (27–31). This method is often associated with the US law cases Frye v United States (1923) (32) and Daubert v Merrell Dow Pharmaceuticals (1993) (30, 33). This binary approach has been debated in the past and has its own problems (33–37). Legal experts are transformed into “gatekeepers and evaluators” of scientific evidence for the court (32, 38, 39). This requires a certain knowledge base, particularly because some of these criteria seem to be difficult to understand and to apply (30, 38, 40). In the American legal system, some judges have taken it upon themselves to become “amateur scientists.” They participate in training courses on scientific methodology and principles in an attempt to increase their scientific literacy skills and become more familiarized with scientific terms and methods (5).

Where admissibility of evidence is not officially regulated by criteria, the other approach is to hope that the usual trial procedures will weed out inadequate testimony (33). Expert training, mandatory special instruction and certification (41) or peer-review of expert testimony (42, 43) are meant to insure high- quality expert testimonies and make them more reliable. Efforts have also been made to improve the communication between scientific and legal experts, for example by standardizing written testimonies and adapting them to legal experts' needs (44–47). The use of impartial experts appointed by the court (48, 49) or employed by the state (50) as well as defining codes of conduct for experts (46, 51) are attempts to reduce potential expert bias. Furthermore, allowing more than one expert witness to address the court (52) helps to avoid relying upon one single, potentially flawed statement.

These measures may help narrow the knowledge gap by weeding out unusable evidence and ensuring impartial expert witness statements of high quality. However, as stated above, the problem of conflicting evidence remains. If judges and juries are to avoid delegating too much responsibility to a scientific expert for a legal ruling, then it is important for legal professionals to decide in the face of diverging medical statements which one to follow. This arguably entails possessing a modicum of knowledge of the science in question (3, 53), such as the understanding of medical terms and the procedures and limitations of scientific methods. In addition, they need to be able to critically appraise and understand scientific information, one of the definitions of scientific literacy (54).

Previous studies have investigated different aspects of this topic; for example, how judges deal with expert knowledge focusing on the Daubert criteria (38), the characteristics and criteria that are used by judges to evaluate expert testimony (31, 55), how judges evaluate scientific knowledge (56) or behavioral factors of expert testimony (57). However, research is limited regarding experiences and perceptions as to how medical expertise is handled.

Switzerland with its four official national languages German (>70%), French (20%), Italian (<5%), and Rhaeto-Romansh (<1%) follows an inquisitorial legal system similar to those in other European, Asian, Latin American, and African countries. Because of Switzerland's federal structure, the organization of the courts as well as the jurisdiction in civil and criminal matters remains with each of its 26 cantons. Thus, the cantons do not just have a considerable degree of law-making authority, but also have the right to regulate the organization of the courts and their procedures. The Swiss court system is thereby divided into civil, criminal, and administrative courts with a Supreme Court on the federal level, located in Lucerne and in Lausanne (58).

“The procedural law applied by the cantonal courts is thus primarily state or cantonal law (with various incursions of federal law and public international law) while, depending on the area, substantive law can either be federal law (this is the general rule in regard to private and criminal law) or state law (in several areas of public law)” (58). Within this judicial system medical knowledge enters a legal proceeding via the statements of medical experts or specially trained expert witnesses (59, 60), who are often paid and retained by public agencies. It is also possible for legal experts to base their argument on their own resources, such as books or internet websites. The principle of free evaluation of testimony allows judges to decide which evidence or argument to follow.

Few studies exist regarding legal experts' experiences in obtaining medical knowledge and scientific literacy skills and the resources used. It could be argued that since forensic science exists somewhere between the disciplines of medicine and law, it is well-placed to further knowledge on how to improve the legal system with respect to medical knowledge. Here, we propose to explore these gaps by undertaking a qualitative interview study, which aims, firstly, to describe legal experts' experiences of medical-knowledge gathering and acquisition and attainment of scientific literacy skills for professional purposes. We focus in particular on experiences during and after law school. Secondly, we aim to describe what tools legal experts perceived to be useful for learning and understanding medical terms and assessments.

## Methods

### Study Design and Sample

A qualitative study design was considered appropriate for the exploratory nature of the research. Qualitative research is employed across different disciplines, including medicine, in order to understand phenomena, people's experiences, perceptions, attitudes, and the meanings they assign to them (61). In the field of forensic psychiatry and psychology, for example, this methodology has helped to identify elements and conditions that are perceived to be helpful by all parties involved in the therapy of sex offenders (62), to examine descriptions of maternal filicide committed in the context of major mental illness (63) and to investigate high-dose benzodiazepine dependent patients' perceptions and beliefs surrounding criminal and violent behavior and insight, both before and after episodes of memory disturbance (64). Our data regarding the legal experts' experiences and perceptions was thus collected by conducting semi-structured one-to-one interviews.

A purposive sampling procedure was used for participant selection. “It is typically used in qualitative research to identify and select the information-rich cases for the most proper utilization of available resources. This involves identification and selection of individuals or groups of individuals that are proficient and well-informed with a phenomenon of interest” (65). To achieve greater variation of themes and motives, we recruited subjects from different courts of law and from the public prosecutor's office in six different German- (Zurich, Luzern, Aargau, Obwalden, Nidwalden, Zug) and German/French- (Bern) speaking cantons of Switzerland. Furthermore, the sample was chosen to incorporate diversity with regards to: (a) work experience, (b) legal focus, (c) gender, (d) age, and (e) seniority of position. Seniority was thereby defined as being at least the president of a court or the head of department in a public prosecutor's office. As mentioned above, Switzerland is a federal state, in which each canton has its own constitution, legislature, executive (police) and courts. For this reason, despite their equivalent roles in the cantons, the professional titles of the participants varied from canton to canton. Exclusion criteria were unwillingness to give written informed consent.

The research team pursued multiple strategies to contact potential participants: (a) Individuals who appeared to be especially “information rich” (e.g., a former male judge of the Swiss Federal Court, a female judge with a previous teaching and publication history) were contacted either by phone or in person and asked to participate; (b) A local information session in the canton of Bern and a regional congress in Zurich were used to inform judges and public prosecutors about the study; (c) an opt-in letter (429 words) was sent to the departments of prosecutors and judges in different German- and French-speaking regions of Switzerland, inviting them to participate. Candidates who expressed an interest in the study were approached privately by a team member, via email or telephone, providing them with more details regarding the research and answering their questions. The latter approach was undertaken to broaden the spectrum and thus to include candidates in junior positions and those less likely to participate in medico-legal meetings.

Recruitment was ceased after saturation had been reached. Saturation is commonly defined as the point when no new themes arise. The subjects provided additional basic biographical data.

### Procedure

We used a self-developed, semi-structured and flexible interview guide to explore themes of knowledge acquisition among legal experts. Before the interviews, participants were notified that: the interviewers had a forensic psychiatry background and the research would address the collaboration between legal and medical experts. Open-ended questions and non-leading probes were used to encourage participants to speak freely and to elaborate on their statements. Paraphrasing the participants' answers helped clarify ambiguous statements. The interviews covered several topics that were beyond the scope of this manuscript but will be reported on subsequently.

The interviews were conducted by four researchers (CC, IP, RN, ML) in the following languages: Swiss German in the German-speaking parts of the country, French in the francophone parts of Switzerland and in some cases Standard German. Additionally, field notes were completed for all interviews. All interviewers were psychiatrists (i.e., MDs) with experience and training in forensic psychiatry and qualitative research. Previous research results employing qualitative methodology can be accessed using Google Scholar (66). At the time of the study all interviewers were employed in forensic institutions. Interviews were conducted on a one-to-one basis and were digitally recorded. Careful attention was given to establishing rapport and minimizing interview bias (67). The interview's sequence, pace and duration (between 45 and 90 min) was participant dependent. By grounding the questions in participants' legal training and practice experiences, and by reformulating the questions, we sought to avoid idealized, generalized, flippant and/or tangent responses. By allowing participants to have control of the interview location, pace and duration, we sought to create an atmosphere which allowed for eliciting more “private” opinions and experiences. With the exception of two interviews that were conducted in the office of IP, all other interviews were carried out at the subject's workplace. There were no repeat interviews.

Each interview was transcribed verbatim in its original language (German or French), except for those interviews in Swiss-German, as these dialects do not have a written form. For these interviews transcription was completed in standard German. Each interview had a code number assigned and all potentially identifying information was removed. The transcripts were not returned to the interviewees.

**Figure 1:**
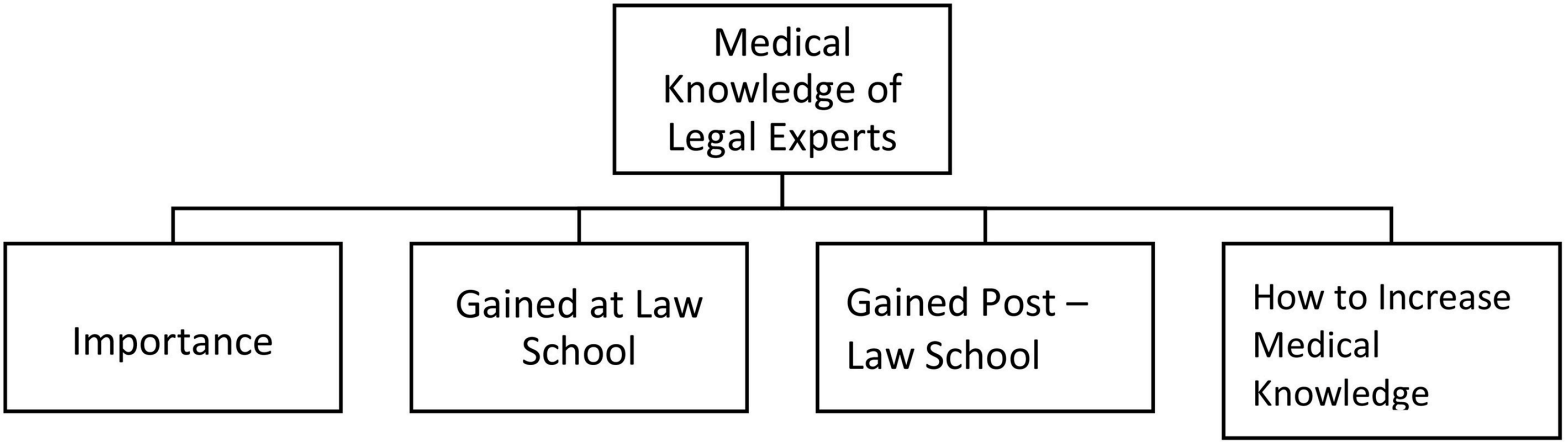
Main categories.

The same four interviewers undertook the qualitative analysis of the interview data independently. We used a comparison thematic approach, identifying common and new themes related to the research aims. For this research, the interviews were analyzed with QSR NVIVO 11 for Windows, a qualitative data analysis software (QDAS) (68). This software was used primarily as an organizing platform and as a tool to illustrate the coding tree (see Figure 1). Coding centered on identifying common and unique themes related to the research aims, as well as omissions within the interview transcripts.

The coding process ensured a systematic, comprehensive and detailed reading of each interview transcript. First, the coders familiarized themselves with the transcripts in order to identify the different subjects of interest to the forensic field. After several interviews had been coded, the categories for the study were redefined, reviewed and revised in a consensual manner at regular meetings. When there was disagreement regarding the code or coded material, ML applied the final code. The additional/new themes led, in accordance with qualitative research methodology, to an adaptation of the topic guide and to supplementary telephone interviews in 29 cases. Five interviewees requested that the additional questions be posed in writing and answered by letter. As a result of the coding process and the purpose of this report, four main categories were identified and selected: (a) importance of medical knowledge, (b) medical knowledge acquisition at law school, (c) medical knowledge acquisition after law school and (d) strategies for increasing knowledge. An overview of the categories is shown in Figure 1.

To illustrate the categories and for reporting purposes, exemplary coded quotations were chosen by CC and translated from German or French into English. Google translate was used to translate interview quotes in our findings (all in German) into English for publication purposes. These were then discussed and improved by a native German speaker and an English native speaker to ensure readability and accuracy.

The research aimed to capture all the possible experiences and perceptions which were collected in the textual narratives and opinions of judges and prosecutors. However, in order to orient the reader regarding the prevalence of similar opinions among the interviewees, as perceived by the coder, a summary statement is reported, identifying the number of participants whose interview transcripts referred to the theme discussed. Since the interviews developed differently, reporting a percentage or weighting of certain statements would be potentially misleading. Moreover, statements of implicit agreement were not counted. Nevertheless, in Annex A, a summary table with theme and frequency information is presented.

This investigation is reported as having adhered to the 32-item Coreq comprehensive checklist for qualitative research (61). This checklist was specifically developed for reporting qualitative interviews and “covers necessary components of study design, which should be reported” [(61), p. 356]. By complying with these guidelines, we ensure that essential aspects are described which allow the reader to assess the study's validity, such as: the research team, reflexivity, study design, data analysis and reporting.

Zurich's cantonal ethics committee filed a letter of non-competence, stating no objection. All participants were assured confidentiality, and gave their written informed consent to the study and, specifically, to the digital recordings of the interviews obtained.

## Results

### Sample Descriptions

The research team was in personal contact (face to face, telephone, or e-mail correspondence) with 67 potential participants. Fifteen declined to participate. Barriers to participation were rarely addressed spontaneously. Four possible participants refused on the grounds that they were generally not available for studies. Five declined to participate, citing a lack of time due to high workload. Six gave no reasons. Thus, 52 interviews were conducted. After conclusion of the interviews, one participant withdrew their consent and consequently their interview was removed. One participant unexpectedly died between the personal and phone interviews and could thus not voice his opinion on supplementary questions, however his primary interview was included. In a few instances, potential participants had questions about anonymity or expressed a desire to obtain permission from their supervisor. However, in no case did these concerns lead to non-participation.

In total, 51 subjects provided their written, informed consent and completed the interview. The sample (*n* = 51) was composed of a slightly higher percentage of judges (53%) than of prosecutors (47%). The sample included assistant-prosecutors, prosecutors, senior-prosecutors, heads of the department of the public prosecutor's office and court clerks, presiding judges of regional/district courts, higher cantonal courts as well as a former member of the federal court. All 51 legal experts reported having experience in criminal law. Further, some reported experience in other legal sectors, such as civil law (*n* = 12), family law (*n* = 5), defendant (*n* = 1), and all law fields (*n* = 1).

Of the participants, 29 were identified as holding senior positions, according to our definition. Male legal experts represented 66.7% of the sample, females 33.3%. Their age ranged from 29 to 73 years (*M* = 49.96 years and SD = 11.29) and average years of employment ranged from 5 to 49 years (*M* = 22.71, SD = 10.76).

### Importance of Medical Knowledge

**Figure 2:**
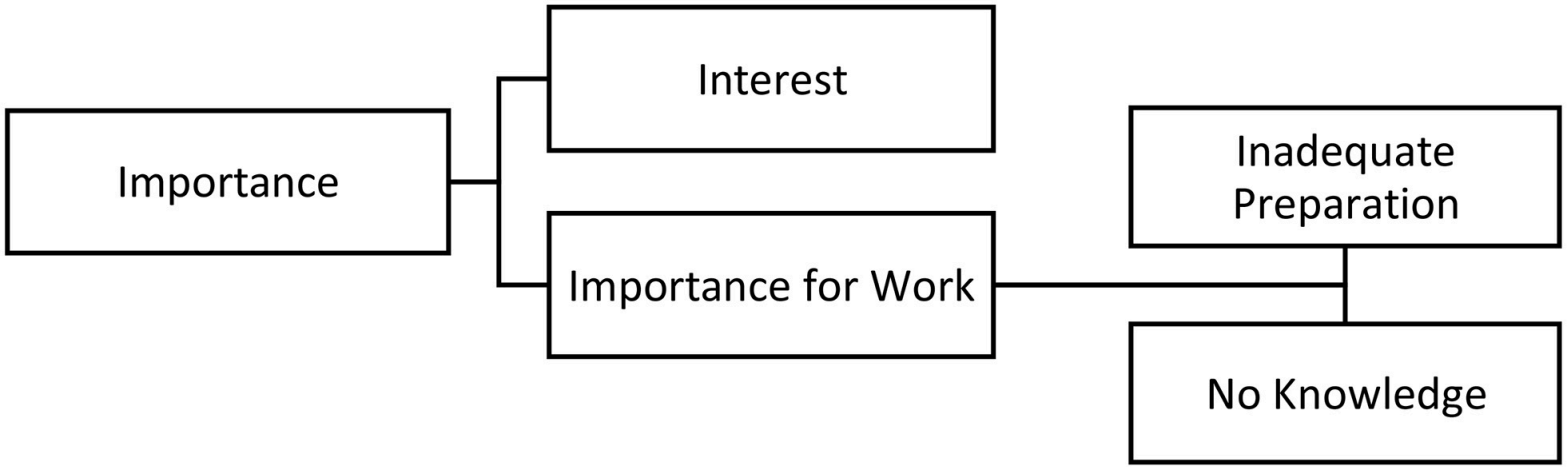
Importance of medical knowledge.

One of the first themes to arise was the value the participants accorded to medical knowledge for their legal practice (Figure 2).

Participants (22) expressed in their own words how medical knowledge was important for their work. They referred to medical assessments as being a very important component of cases they judged or represented. In addition, legal experts (38) professed to have a special interest in medical knowledge. Participants (5) recognized that cases requiring medical knowledge were more time-demanding and complex compared to cases requiring only legal knowledge.

…*yes, very important, was an injury life-threatening or not, this is crucial…(VP15)*

…*Well, I am interested, very interested, yes, it's a really important aspect (…) of course the repercussions are considerable, especially for the consequences if criminal behavior is proven, or whether there is a therapy or not, and, yes, there is also the question of culpability, which can be very important… (VP19)*

…*In 10–20% of the cases, yes, there is the core issue… (VP12)*

…*Compared to the other cases, these [dealing with medical information] require much more time, because they are usually more complex… (VP13)*

Legal experts (11) expressed awareness of the importance of scientific knowledge, often coupled with the perception of being inadequately prepared to deal with such knowledge. This knowledge gap appeared to one of the legal experts to justify delegating part of the responsibility for legal solutions to scientific experts.

…*I mean, we have so many fields we should know about, you know, if you think about building law, I have to rely on experts there as well, I just can't evaluate this myself, sometimes I don't even understand what the problem is, it just comes in like that and then the experts deal with it and resolve it and it's the same with medicine. It would certainly be great to know a lot about it and the newest scientific results…(VP16)*

Legal experts (11) clearly expressed their lack of knowledge in this area.

…*my medical knowledge is like zero, nonexistent…(VP24)*

I have relatively little, in fact, I have absolutely no sound medical knowledge… (VP7)

### Medical Knowledge Acquisition at Law School

**Figure 3:**
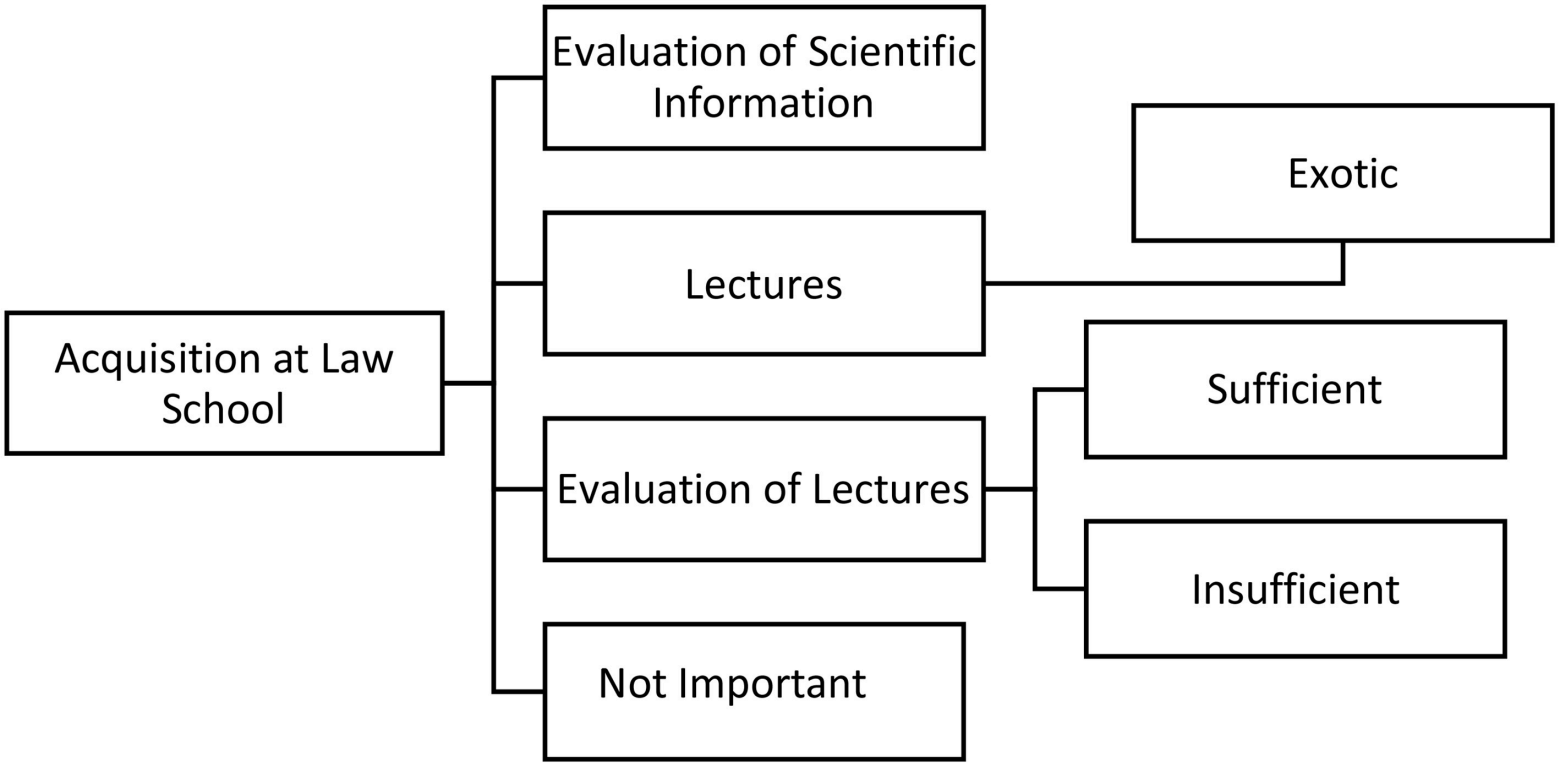
Medical knowledge acquisition at law school.

None of the participants reported having specifically learned how to evaluate the quality of medical information (Figure 3).

A clear majority of participants (37) claimed not to have been specifically taught any medical knowledge or scientific literacy skills during their undergraduate degree.

Res.: During your training, or when you were at law school, was the evaluation of medical knowledge ever a topic? Did you learn how to deal with expert testimony or medical knowledge?

B: No, not at all. (VP2)

Res: Was the evaluation of medical knowledge ever a topic in law school or later, in your postgraduate career?

B: I don't know, now, where you are going with this, but I would say no. (VP12)

Res.: Did you notice a subject on how to evaluate medical knowledge at law school?

T: During law school I attended a course on forensic medicine. Yes, it was very interesting. I was also able to attend a course once about psychiatric diagnosis. In that one, we were confronted with different people, like people with schizophrenia, or as I remember, dependency was also a topic, alcohol, drugs, but not chiefly. These were extracurricular classes, to be attended voluntarily. It was not mandatory. (VP25)

Participants (4) who received training in forensic medicine reported having difficulties in assimilating those courses in a useful manner. Instead, they (3) perceived such lessons as exotic or entertaining, rather than practical.

I took an optional course about forensic medicine, once for a couple of hours, but no, when I graduated I had no idea that there even was such a subject like forensic psychiatry (laughs)…(VP1)

No, in law school, no, I mean there was of course a lecture on forensic medicine, but it was just a marginal subject, really… (VP14)

…*we went to the Institute of Forensic Medicine, they were conducting a clinical autopsy, the way a corpse looks, that's rather, eh, that's nearly a bit like voyeurism… (VP8)*

Interviewees (8) considered that the medical knowledge they were taught at law school was insufficient and felt that it had poorly prepared them to deal with it in a professional context. Only one respondent recognized this as a limitation of their training. However, four participants regarded training in scientific literacy skills and skills in reviewing medical knowledge during law school as not relevant at the time.

…*At the time I started, that wasn't important—it wasn‘t an issue…(VP15)*

…*During law school not at all, this was not an issue for me during law school… (VP24)*

…*retrospectively I have to say that this was a shortcoming [during law school]… (VP43)*

In hindsight, others (9) regarded their training in medical knowledge at law school as sufficient. They considered it more useful to acquire knowledge in this field at a later stage of their career, particularly after they had acquired some first-hand experience. Participants (3) believed that such knowledge was only necessary in criminal law and because relatively few legal professionals practice this field of law, it was not advantageous to teach it at undergraduate level.

I don't think it makes sense either. I believe you need a certain work experience for this matter. At least, you need to have dealt with those people and experienced them, gotten to know them a bit… (VP13)

…*in my opinion it makes sense to acquire it later, in the field, because you also have to see, that there are only a few working in criminal law. If you look at the students at law school, there are only a few that choose criminal law…(VP38)*

### Medical Knowledge Acquisition Post Law School

**Figure 4:**
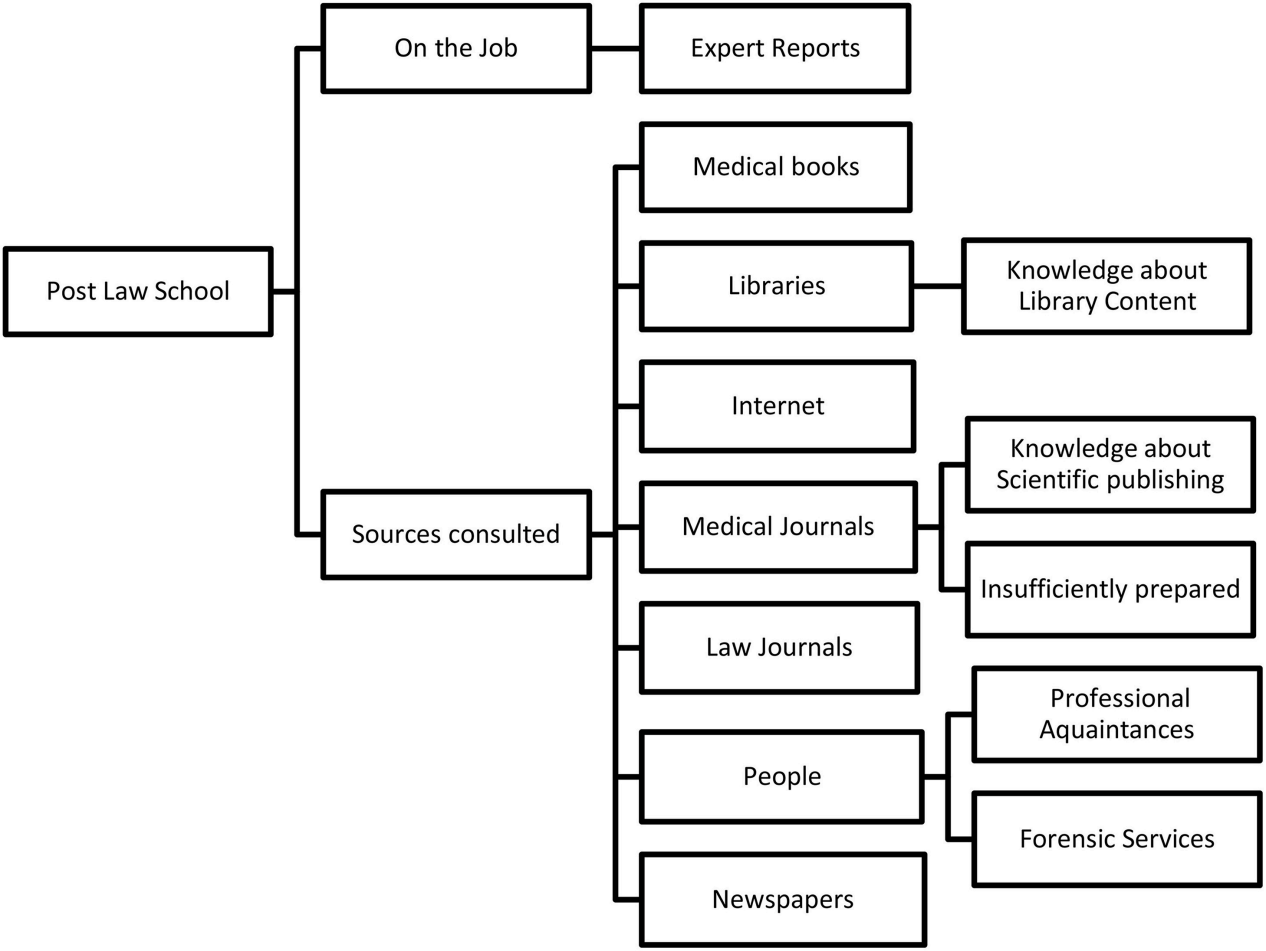
Medical knowledge acquisition post-law school.

Participants mentioned attending postgraduate medical-legal courses and conferences. Legal experts (20) described learning medical knowledge “on the job” and from medical expert reports (Figure 4).

Therefore, it appears that their knowledge acquisition resulted from self-directed and informal learning. Others explicitly stated they had not attended a postgraduate interdisciplinary course.

I didn't learn my medical knowledge anywhere specific. It was just learning by doing. (VP10)

One source is certainly that we are confronted with case files in our everyday work that deal with certain topics, so we observe certain case patterns and you can also see them in the medical expert reports, eh, and of course, the more inexperienced you are, the more you pay attention to the terms in the reports, you gather the definitions, eh, this way you accumulate the information, just from these reports. And if you have the occasion to have a medical expert present at court, you use this occasion to ask him additional questions…(VP12)

Interviewees did not know whether medical literature was included in their library at work and others stated that their libraries held only a few medical books or none at all (14).

*Yes, we have the Pschyrembel* (author comment: a well-known medical encyclopedia in German) *maybe, I have one of my own, but otherwise we don't have many about medicine… (VP27)*

Res.: Can you tell me what medical, forensic, psychological literature there is?

P: No, I can't, I never looked. (VP33)

Interviewees reported not consulting medical books at all. Those (14) that did, reported using the diagnostic manual, a pharmacological compendium, a medical encyclopedia and sometimes a forensic medicine or psychiatry textbook.

I acquired my knowledge, yes, formerly from the Pschyrembel, which was the only manual, so the manual which one consulted before the Internet came up and nowadays from the ICD… (VP20)

All judges and legal experts (51) reported researching medical terms using the internet, chiefly Wikipedia. They used websites for basic information related to diagnosis, prognosis, and treatment.

I use, I know Google and then I look it up there, and see what comes up… (VP8)

I type it in Google and usually Wikipedia comes up with an answer (VP48)

Google, of course, it's normal to sometimes look up a term when you are reading an expert report or when I see that a client needs a certain medication. Then I look it up on the internet, to see what it's used for… (VP42)

Concerning scientific/medical literature, 41 legal experts stated that they were not familiar with any medical journals. Furthermore, they were unaware of relevant databases such as “Medline,” “PsychINFO” or “Google Scholar” and/or terms associated with “evaluating” scientific contributions based on bibliographical data such as “number of citations” and “impact factor.” Even though references to specific journal articles in expert witness reports are not uncommon, they were unable to access those. Only one respondent spontaneously provided the name of three journals and another appeared to recognize a journal the interviewer had mentioned. Interestingly, open access journals were considered to be of lower quality than subscription journals—a conclusion that may have to do with experience in legal publishing.

Res: Do you know the term impact factor?

P: I have heard about it, but I couldn't say in what context… (VP46)

Res: Do you know Google Scholar?Pubmed?

P: No… (VP1)

…* from the name, I know the British Medical Journal, but, so, I don't claim to have ever looked up anything in it; so in this respect it's useless, isn't it?…(VP17)*

…*I don't consult medical literature, because eh, because I can't really read it…(VP1*

Access to medical journals is restricted at our workplace, because we haven't subscribed to any… on the internet there are only a few, and usually they (open journals) are in another language, usually in English and they are probably not the most renowned… (VP39)

So, there are American and English sites, the Lancet or the Journal of Medicine or the International Association of Psychiatrists. In principle, these are interesting sources, anyway. (VP51)

Participants (4) stressed that they considered themselves ill-prepared to read “proper” medical articles. They acknowledged that medical experts required many years of training and there were limitations as “a layman” in regards to what medical knowledge they could understand.

It is relatively useless looking up medical literature without a couple of years of training and knowledge and to say… that is as if you gave a layman a law book and said,” yes, there is something there that fits.” If you have no idea about the system and the context, you should leave it be. Knowing that, I also let it be… (VP17)

Instead of journals, specialized medical services (forensic medicine or psychiatry) with ties to the law were identified by 3 interviewees as resources to be consulted regarding simple questions which do not require an expert statement. Additionally, legal experts (19) believed they could approach their acquaintances and relatives with medical training to obtain medical information (e.g., medical terminology).

I can call your service…(VP51)

…*I can approach my family doctor, with whom I'm on a first-name basis, yes, I could certainly approach him… (VP46)*

My wife is a nurse… and she also did psychiatric nursing and she has got medical books, and I can look something up there or ask her. (VP44)

.…an acquaintance of mine is a general physician, eh, is going to be, and I asked her once…and with another, a psychologist we also once discussed certain topics…(VP41)

Participants (3) considered the newspaper a source of medical information.

I read as much as possible in my free time, the newspapers and then I like to read sometimes, what they write about forensics, that I would do… (VP1)

### Strategies to Increase Knowledge

**Figure 5:**
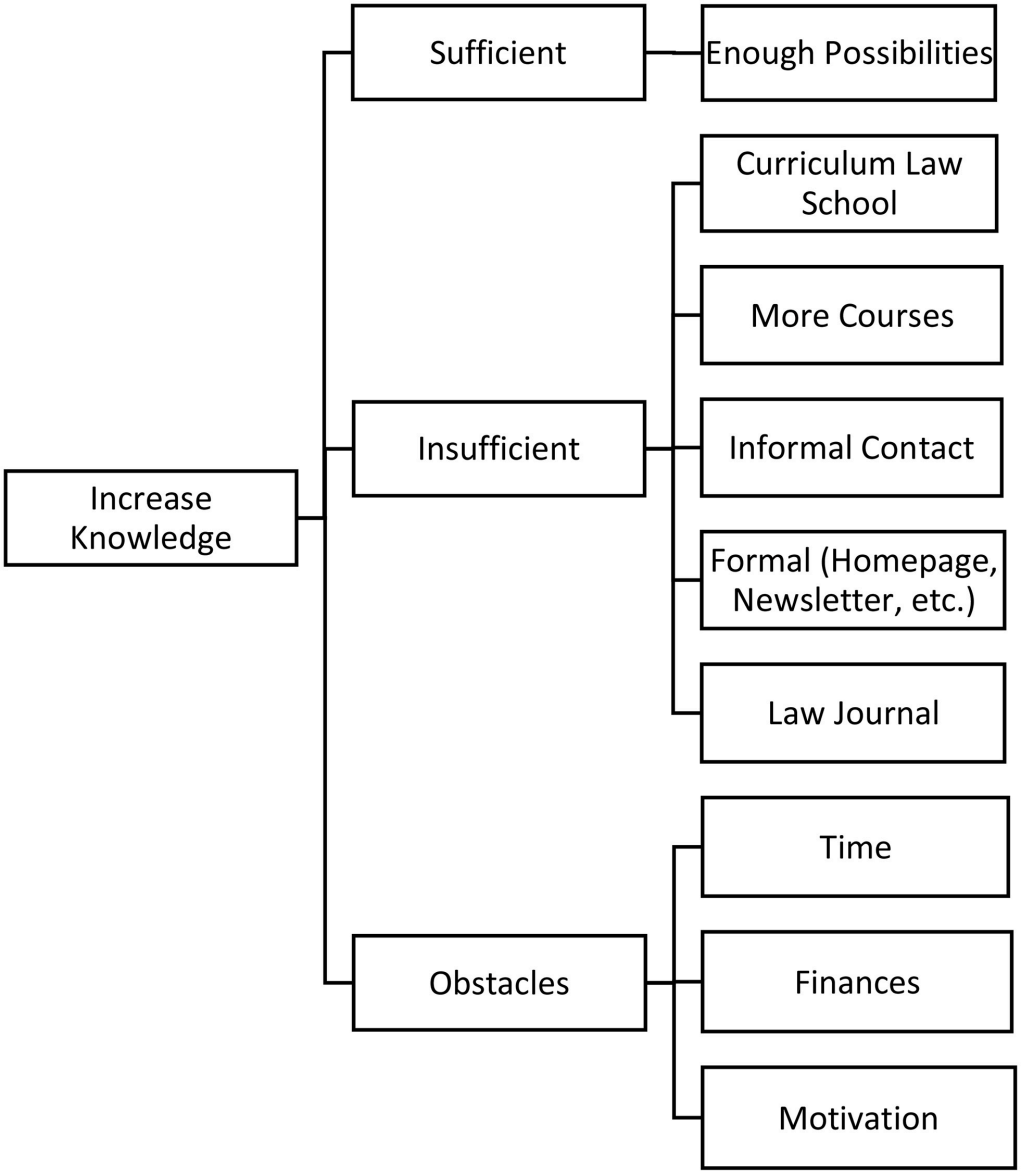
Strategies to increase knowledge about medicine.

Some of the interviewees (18) wished for more opportunities to acquire medical knowledge (Figure 5).

Several participants (5) advocated changes to the law school curriculum, while others (8) identified short courses and events (e.g., conferences, workshops) as useful ways to learn more about medicine.

I think in the context of congresses or conventions, these events already exist, where you truly learn something, where you can perhaps gain new insights…(VP16)

Precisely this interface of psychiatry and justice hasn't been sufficiently developed in postgraduate education. We need to do more…(VP24)

Case discussions are for me [the best]…people learn with cases. And the jurists, they like to have cases. Even the psychiatrists are similar in this respect. (VP44)

That would perhaps be an approach, during law school, to place an increased emphasis on this intersection between medicine and the judiciary. (VP14)

I am struggling to get universities to offer the subject, well, as a “serious” major subject with the corresponding credits, uhm so that the training…so further training is promoted in this area…(VP35)

Some of those (3) who felt that improving the prospects of learning more was unnecessary, were also of the opinion that there were already plenty of opportunities and resources to gain knowledge—e—if only they were consistently used.

I think not. We can get the knowledge, we can recall it. It just has to be done…(VP3)

Other participants (3) wished for more informal contact with forensic institutions. Interviewees (4) suggested alternative means of accessing medical knowledge, such as a specialized hotline, web pages or an electronic newsletter with short articles. Easy access and comprehensive information were valued as significant characteristics for the proposed medical information sources.

I think something that is really easy to access, on the internet or on an information platform, but it should offer an easy entry into and a good overview of the matter, that's something I imagine might be used when questions arise…(VP17)

Actually, the easiest way would be an info-letter, so if it were done by email, done like a newsletter, I think that would be the easiest and most effective way, because lectures [and] conferences, are very focused on a topic, and often go far beyond what we really need to know.… (VP18)

Well, precisely the exchange, for me that's what is exciting, that you have the network, that you can check to clarify, or even ask new questions, if you encounter difficulties with an area…(VP41)

Perhaps in the form of a journal, that one could then subscribe to and read, but I think those [legal professionals] who are interested, they also read this; or in the form of an email, like a monthly newsletter or something like that, or also… that we would have a text book or something, that could be looked into if interested… (VP10)

Time and financial constraints were the most commonly mentioned obstacles to learning medical knowledge among the judges and lawyers (13). In addition, lack of motivation was identified as a barrier to learning by only one participant. While one public prosecutor perceived that courses should be obligatory for all participants, as a means to improve professionalism and preclude a complaint attitude among colleagues.

…*because of the money, the government has not got that much money… (VP23)*

…*Time is also an issue, in practice we have a lot to do and lack the time needed… (VP27)*

I am pragmatic, eh, if I had the choice between going to a two day long conference or getting the essence in written form, in two pages to be read, then, eh, I would prefer to read it, because it takes less time and I would still be informed. If we were to be informed in a brief and concise way by newsletter, or by a source where we could also ask questions when we need to. (VP7)

I believe, or, if you do it on a voluntary basis, as I do. I am now going to Interlaken. Only those who are interested in this topic will go. And uh… the question is: how do you reach those who always complain about medical reports, but actually need it [medical knowledge]? …Uh, and I believe it is really in the interest of our profession and thus also of the employer, that is, the prosecution, to require that participation in further education is compulsory… (VP8)

While reading related medical articles was deemed a good way to access new information, legal experts (8) claimed to only read law journals and were positively disposed toward medical articles in law journals. Furthermore, participants (12) explained that they preferred reading in their native language to reading in English.

That would be, if you have any exposure to the Swiss Journal of Criminal Law or Criminology [Journal], etc. (enumerates several Swiss legal journals), is something I also consider very good. That would also be read by jurists…(VP34)

Res.: Do you have any ideas, or wishes, about how we could make it easier for legal experts to access medical knowledge?

P: I would do it by publishing and offering further education courses. (VP12)

Yes, because the other thing is, I have little time anyway and reading such topics and in a foreign language that really takes up too much time… (VP14)

German, definitely. Medical [literature] is a bit time consuming, so it would be preferable in a familiar language. (VP19)

## Discussion

As far as we know, this is the first qualitative study to investigate how judges and prosecutors acquire medical knowledge, among other things to help them answer legal questions directly or to better understand and evaluate expert witness statements. As this is not quantitative research, this study is not an investigation of “phenomena via statistical, mathematical or computational techniques” but is, rather, intended “to provide a description and understanding of a situation or behavior” (69).

Out of 67 potential subjects, 51 agreed to participate in this study and findings were drawn from the transcripts of these interviewees. Since fifteen subjects chose not to participate, our findings might reflect a volunteer bias, i.e., that legal professionals with a special interest in the intersection of law and medicine were more likely to participate in this study.

In line with the existing literature, our results indicate that legal experts deem medical knowledge to be important for their work and reported depending on outside information, for example, in the form of expert testimony (28, 70). Judges and prosecutors had acquired some general medical knowledge, usually in the form of a lecture focusing on forensic medicine during law school. However, these lectures were viewed as something rather exotic and not necessarily helpful for daily practice. What is more problematic is the finding that none of the participants had consciously learned to evaluate scientific knowledge, which appears to be consistent with the findings of earlier studies involving other legal systems (56, 71, 72). From their statements it could be inferred that legal experts acknowledged requiring scientific knowledge for their legal practice, but not all participants expressed a conscious need to learn how to evaluate scientific information.

They were aware that postgraduate courses and lectures dealing with medicine and law were available. The barriers to training that would increase their scientific literacy skills and knowledge of medical terms for legal practice were lack of time and/or financial costs.

As was to be expected, the internet played an important role in gathering information and appeared to have superseded the use of books. Participants followed a common trend among internet users reported in the past decades (73, 74). Wikipedia was the most cited webpage (75). This is also consistent with previous research findings demonstrating that Wikipedia has become one of the most widely used online sources for medical information by the general public (76).

Although the internet was identified as a popular and useful source of medical information, two problems with or limitations of its use are identified. Firstly, there is the issue of users' ability to find pertinent information on the internet. Secondly, there are concerns regarding quality, as the information available can be highly heterogenous (77) and users' ability to evaluate the information might be not be good enough to allow understanding (78). On the other hand, research indicates that current search engines tend to find websites with medical information of sufficient quality (79). In this case, the quality of the information found may indeed be satisfactory. Nevertheless, the fact that most participants favored Wikipedia (a non-peer-reviewed resource) suggests that legal experts may have insufficient knowledge regarding quality markers for scientific information, for example authorship (80) and peer reviewing. While “bibliometrics” as an indicator for research quality have been under increased scrutiny over the last couple of years (81), a basic understanding of “impact factor” and “number of citations” might help legal experts to assess whether a scientific work cited in an expert opinion represents an individual opinion or is supported by a broad majority of scientists. This differentiation is thereby of great legal importance. The Swiss Federal Supreme Court, for example, has repeatedly pointed out that the source and method of medical knowledge has to be “widely recognized by researchers and practitioners of medical science” (Judgment of the Federal Insurance Court U 160/98 of 2 June 2000, E. 5 and 6 with references, publ. in: BGE 134 V 231 S. 233).

It may be that in the Swiss legal system, where expert witnesses are highly trained and are usually paid by the state, and privately retained experts are the exception, the usual practice of referring to only one expert testimony per case misleads the legal expert into believing the expert statement must be correct. A part of the responsibility for the quality of expert evidence is shouldered by the experts themselves, an attitude consistent with the authors' experiences, as expert witnesses. This could explain why legal experts in an inquisitorial system—or at least in the Swiss system—may feel less pressured to learn how to evaluate medical testimonies or to use criteria compared to their professional peers in adversarial system countries, like the USA (Daubert, Frye). This is problematic, because the quality of expert testimony has repeatedly been criticized in the past, despite the special training medical experts receive before acting as expert witnesses (23, 25, 26, 82–85). On the other hand, it has been shown that the same scientific evidence can be differently interpreted by medical experts (15, 16, 86), an aspect that also finds a parallel in forensic psychiatry. Various studies have shown that the interrater reliability with regard to the diagnosis of mental disorders using the ICD 10 or DSM V is far from perfect (87, 88). However, the specific diagnosis is the “entry criterion” for further medical and legal considerations, including that for therapeutic measures. Thus, disagreement between raters has crucial consequences (89). It seems clear that legal experts should be aware that expert opinions may differ in quality and content and that they should try to understand and, if possible, evaluate them. To achieve this, legal experts would require training on how to handle medical knowledge and scientific literacy skills. Scientific literacy has been defined as the capacity to use scientific knowledge, to identify questions and to draw evidence-based conclusions in order to understand and help make decisions about the natural world and the changes made to it through human activity (90). This definition draws attention to the importance of using scientific knowledge for decision making (91).

Obviously, legal experts are not expected to acquire the same medical knowledge as medical expert witnesses. However, it seems necessary for them to understand medical terms and data to a sufficient degree in order to be able to assess an interpretation, and, preferably, the risks of bias and unreliability in medical forensic evidence. This means having the skills to access and collect pertinent medical knowledge, which in turn includes scientific literacy. Our findings indicate that legal experts acquire and access medical knowledge in an unstructured way, and may also use problematic sources like newspapers, unspecialized medical professionals or non-peer-reviewed sources available on the internet. No participant indicated having formalized, specialized contacts like medical advisors or links to medical forensic specialist associations, despite acknowledging these as potentially useful sources. Respected academic medical journals and books appear to be insufficient means for bridging the interdisciplinary gap between medicine and law. It would appear that forensic articles published in medical journals dealing with interdisciplinary topics of possible interest for legal experts—for example the aforementioned articles about quality problems of expert witness statements—have a negligible chance of actually being read by legal experts.

### Limitations

These results need to be considered within the limitations of the investigation. First, because this is an exploratory qualitative study based on a purpose sampling method, the findings cannot be generalized beyond this study sample. Second, there are limitations associated with volunteer bias, to which most studies are also susceptible. The main reason for non-participation stated was time limitation due to lack of time and workload. However, other possible reasons could include lack of interest in the subject matter, or sensitivity regarding the topic. We only interviewed members of two of the three sides of a law case as defending lawyers were not included. Thus, these results may not reflect the attitudes of such experts nor of recently graduated lawyers. As exploratory research, this study was not driven by a theoretical framework. Future studies on this subject could, however, use the insights gained to pursue more focused research.

We also recognize that the results may in part be specific to the Swiss legal system. Nevertheless, the literature indicates that similar problems, such as difficulties in evaluating scientific knowledge can be found elsewhere (27). Furthermore, we interviewed prosecutors and judges in different job positions, but were only able to interview one former (retired) member of the federal court, the highest court in the Swiss system. This court's rulings strongly influence policies and it is possible that its judges work differently than most of our participants and have better access to medical information. However, our findings provide several relevant insights into resources and strategies that could be employed to help legal experts. Most importantly, our findings are based on legal experts' own reports identifying a range of experiences. These findings were not limited to predefined experiences, as might occur in a survey-based research. In order to ascertain generalizability, future research could consider studying legal experts' experiences by employing a quantitative design with a random sample. Such a study could also investigate what variables such as age, gender, seniority of position or legal field predict scientific competence and/or access to medical evidence of legal professionals.

## Conclusions

This research extends our understanding of legal experts' perceptions and experiences of the acquisition of medical knowledge and scientific literacy skills for their profession. Findings have shown several different strategies that legal experts undertake when required to understand and assess medical content drawn from expert witness statements. In light of our findings, several practical implications can be drawn. It may be inferred from our results that for medical knowledge to reach legal professionals, it has to be presented in a way that is comprehensible to the layman and, when possible, in their native language. Moreover, it should be presented where they would normally seek information, such as legal or interdisciplinary journals. Many participants professed a need for direct contact with medical professionals, be it their own acquaintances, a speaker at a conference or employees of forensic institutions. As no interviewee reported having formalized specialized contacts with medical institutions, offering easier access to forensic institutions would enable better interdisciplinary cooperation and further the quality of knowledge on which legal experts can base their decisions.

Forensic science professionals, legal experts, legal clients and the general public would naturally benefit from legal experts acquiring the necessary tools with which to evaluate medical witness statements. Scrutiny of forensic evidence would be improved if lawyers acquired the scientific literacy skills enabling them to ask pertinent questions regarding the reliability and biases of data and of expert conclusions in medical expert statements. Ultimately, such scrutiny would help legal professionals to better achieve the goal of legal equality and legal certainty.

In the light of our findings, law departments might wish to evaluate whether their graduates are sufficiently equipped with scientific literacy skills. In addition, courses on internet use as well as on evaluating internet resources and information are extremely important and teaching those topics formally at undergraduate and postgraduate level should be considered, as well as in specific short courses. Finally, forensic research into the reliability and bias issues of medical forensic evidence should be increased and communicated not only to peers in the field but across disciplines to legal experts.

## Author Contributions

CC: data gathering, evaluation of data, writing, and revisions. AB: conception, development of the topic guide, and writing. AD and CJ: conception and revisions. IP and RN: data gathering. PS: writing and revisions. ML: conception, development of topic guide, obtaining ethical approval, evaluation of data, writing manuscript, and revisions.

### Conflict of Interest Statement

The authors declare that the research was conducted in the absence of any commercial or financial relationships that could be construed as a potential conflict of interest.
