# Supplementary material for: How Do Legal Experts Cope With Medical Reports and Forensic Evidence? The Experiences, Perceptions, and Narratives of Swiss Judges and Other Legal Experts
Source: Front Psychiatry. 2019 Feb 13;10:18. doi: 10.3389/fpsyt.2019.00018 (PMC6381858; doi:10.3389/fpsyt.2019.00018)
Supplement: Supplementary file 1 [file Table_1.docx]

**Annex A**

**Table 1. Summary of findings**

| **Theme** | N of interviews* |
| --- | --- |
| **Importance and medical, forensic & knowledge acquisition during law school** | |
| Acknowledging the importance of medical knowledge for their profession | 22 |
| Professing having an special interest in medical knowledge | 38 |
| Cases with medical knowledge described as time-demanding and complex | 5 |
| Perception of being inadequately prepared to deal with scientific knowledge | 11 |
| Delegation of responsibility for legal solutions to scientific experts due to knowledge gap | 1 |
| Process (handle) medical information | 51 |
| Expressed having no or limited medical knowledge | 11 |
| Specifically learning to evaluate the quality of medical information during their undergraduate course | 0 |
| Expressed not being taught medical knowledge or scientific literacy skills at undergraduate degree | 37 |
| Difficulties in assimilating medical courses in a useful manner | 4 |
| University medical-related lessons perceived as exotic or entertaining, rather than practical | 3 |
| Medical knowledge taught was perceived as insufficient and poorly prepared them for professional use | 8 |
| Expressed that training in scientific literacy skills and how to review medical knowledge during law school, was not considered relevant at the time | 4 |
| Perception that training at law school was sufficient | 9 |
| Belief that learning relevant medical knowledge should take place after acquiring some first-hand experience | 9 |
| Justifications for not learning applied medical knowledge at undergraduate level | 3 |
| **Medical knowledge acquisition post law school** |  |
| Learning medical knowledge “on the job” from reading expert medical reports | 20 |
| Stating not knowing whether their work library included medical literature  or stating that libraries held only a few medical books or none at all | 14 |
| Using a diagnostic manual, a pharmacological compendium, a medical encyclopaedia and sometimes a forensic medicine or psychiatry textbook | 14 |
| Internet as search tool for medical terms, diagnosis, prognosis and treatment | 51 |
| Lack of knowledge of medical peer-reviewed journals or terms appropriate to scientific/medical publishing (e.g “Pubmed”, “Impact and Google Scholar) | 41 |
| Perception: ill-prepared to read “proper” medical article | 4 |
| Specialized medical services (forensic medicine or psychiatry) with ties to law as sources of medical information | 3 |
| Medically trained acquaintances and relatives as sources of medical information (e.g. medical terminology) | 19 |
| Newspaper as a source of medical information | 3 |
| **Strategies to increase knowledge** |  |
| Expressed desire for more opportunities to acquire medical knowledge | 18 |
| Advocated changes to the law school curriculum | 5 |
| Identified short courses and events (e.g. conferences, workshops) as means to learn more about medicine | 8 |
| Believe that opportunities and resources to gain knowledge already exist but are not consistently used | 3 |
| Desire for more informal contact with forensic institutions | 3 |
|  |  |
| Alternative means of accessing medical knowledge ( specialized hotline, web pages or an electronic newsletter with short articles). Easy access and comprehensive information were valued as significant characteristics for the proposed medical information sources | 4 |
| Time and financial constraints as barriers to acquiring medical knowledge | 13 |
| Lack of motivation as a barrier to learning | 1 |
| Obligatory courses for all participants, as a means to improve professionalism and preclude a complaint attitude among colleagues | 1 |
| Perception and behavior differences regarding medical literature | 8 |
| Language preferences regarding reading sources | 12 |

*Interview guide was semi-structured, so questions were not identical across whole survey, % based on total interview N= 51 is not possible.
